# miRFA: an automated pipeline for microRNA functional analysis with correlation support from TCGA and TCPA expression data in pancreatic cancer

**DOI:** 10.1186/s12859-019-2974-3

**Published:** 2019-07-16

**Authors:** Emmy Borgmästars, Hendrik Arnold de Weerd, Zelmina Lubovac-Pilav, Malin Sund

**Affiliations:** 1 Department of Surgical and Perioperative Sciences, Umeå University, Umeå, Sweden; 2 School of bioscience, Systems Biology Research Centre, University of Skövde, Skövde, Sweden; 3 Department of Physics, Chemistry and Biology, Bioinformatics, Linköping University, Linköping, Sweden

**Keywords:** miRNA functional analysis, miRNA target prediction, Functional enrichment, Mature miRNA, TCGA, TCPA, Pancreatic cancer

## Abstract

**Background:**

MicroRNAs (miRNAs) are small RNAs that regulate gene expression at a post-transcriptional level and are emerging as potentially important biomarkers for various disease states, including pancreatic cancer. In silico-based functional analysis of miRNAs usually consists of miRNA target prediction and functional enrichment analysis of miRNA targets. Since miRNA target prediction methods generate a large number of false positive target genes, further validation to narrow down interesting candidate miRNA targets is needed. One commonly used method correlates miRNA and mRNA expression to assess the regulatory effect of a particular miRNA.

The aim of this study was to build a bioinformatics pipeline in R for miRNA functional analysis including correlation analyses between miRNA expression levels and its targets on mRNA and protein expression levels available from the cancer genome atlas (TCGA) and the cancer proteome atlas (TCPA). TCGA-derived expression data of specific mature miRNA isoforms from pancreatic cancer tissue was used.

**Results:**

Fifteen circulating miRNAs with significantly altered expression levels detected in pancreatic cancer patients were queried separately in the pipeline. The pipeline generated predicted miRNA target genes, enriched gene ontology (GO) terms and Kyoto encyclopedia of genes and genomes (KEGG) pathways. Predicted miRNA targets were evaluated by correlation analyses between each miRNA and its predicted targets. MiRNA functional analysis in combination with Kaplan-Meier survival analysis suggest that hsa-miR-885-5p could act as a tumor suppressor and should be validated as a potential prognostic biomarker in pancreatic cancer.

**Conclusions:**

Our miRNA functional analysis (miRFA) pipeline can serve as a valuable tool in biomarker discovery involving mature miRNAs associated with pancreatic cancer and could be developed to cover additional cancer types. Results for all mature miRNAs in TCGA pancreatic adenocarcinoma dataset can be studied and downloaded through a shiny web application at https://emmbor.shinyapps.io/mirfa/.

**Electronic supplementary material:**

The online version of this article (10.1186/s12859-019-2974-3) contains supplementary material, which is available to authorized users.

## Background

MicroRNAs (miRNAs) are small RNAs of about 19–24 nucleotides [1]. Two miRNA isoforms, termed -3p and -5p arms, are formed from stem-loops that originate from miRNA genes. Usually one of the mature miRNAs, called the passenger strand, is degraded and the other strand, often referred to as guide strand, is playing a role in miRNA-mediated regulation [1]. Nonetheless, both strands may act in miRNA-mediated regulation. MiRNAs are generally considered down-regulators of mRNAs at a post-transcriptional level, but they can also act as up-regulators [2, 3]. In miRNA-mediated down-regulation, translational repression is usually the primary event followed by mRNA degradation [4]. MiRNA-mediated up-regulation may occur indirectly by interfering with repressive miRNA ribonucleoprotein complex (miRNPs) or directly by the activity of miRNPs [5]. Positive regulation seems to be restricted to certain cell conditions, for instance cells in G0 cell cycle state [2].

Pancreatic ductal adenocarcinoma (PDAC) is the most common form of malignant pancreatic neoplasms [6], often diagnosed at a late clinical stage, with very poor prognosis due to early metastatic spread [7]. The most commonly used diagnostic biomarker today is carbohydrate antigen 19–9 (CA 19–9). However, this biomarker has several disadvantages including suboptimal specificity, with elevated levels detected in other diseases, and false negative detections [8]. Hence, research efforts need to be directed towards finding novel, more reliable biomarkers. MiRNAs are highly stable in blood and have been studied as potential non-invasive biomarkers in numerous diseases, including pancreatic cancer [7, 9, 10]. Recently, 15 circulating miRNAs with significantly altered expression levels at PDAC diagnosis were identified and a combination of these miRNA biomarkers was shown to outperform CA 19–9 as a diagnostic marker in terms of area under curve (AUC) [7].

In order to understand the role of miRNA biomarkers, in silico-based functional analysis can be performed, which typically consists of target prediction following functional enrichment analysis of identified miRNA targets [11]. Several R packages and web resources exist for miRNA functional analysis. MultiMiR [12] and RBiomirGS [13] are R packages that perform miRNA target prediction, while RBiomirGS performs functional enrichment analysis as well. The R package MiRComb utilizes miRNA-mRNA expression correlations followed by miRNA target prediction based on negatively correlated targets [14]. MiRLAB performs target prediction and enrichment analysis in combination with mRNA and miRNA expression data provided by the user or from the cancer genome atlas (TCGA) to infer regulatory relationships [15]. Recently, a shiny web application named miRCancerdb was published, enabling users to study correlations between miRNA expression to its targets or non-targets on mRNA and protein expression levels using TCGA data [16, 17]. Another example of a web-based tool is DNA intelligent analysis (DIANA)-mirPath v3.0 [18], which performs miRNA target prediction and functional enrichment generating a list of target genes as well as gene ontology (GO) terms and Kyoto encyclopedia of genes and genomes (KEGG) pathways.

MiRNA target predictions usually generate a high false-positive rate and the most preferable way of evaluating miRNA target predictions is experimental validation [19]. This is however not always possible due to a high number of predicted targets, although databases for collected experimentally validated miRNA targets exist [20]. Validation of identified miRNA targets is a challenge and an intermediate step from prediction to wet lab validation is of great benefit to narrow down interesting candidates. One in silico-based validation approach is to correlate miRNA and mRNA expression levels in combination with miRNA target prediction. A common approach when analyzing the regulatory effect of specific miRNAs is to study changes on mRNA level, whereas regulatory effect of miRNA might in some cases only impact the protein level [4]. In a correlation analysis approach, it is helpful to include protein expression levels since mRNA levels do not always correlate with protein expression levels [21]. Another limitation of some studies is the assumption that miRNAs act as down-regulators of target genes, which is why mainly negative correlation is often considered [22, 23]. As mentioned, positive miRNA-mediated regulation may also occur [2, 3] and hence it is important to also include positive correlations.

Here, we describe miRNA functional analysis (miRFA), a pipeline built in R that provides following features:

1. MiRNA target prediction using two target prediction databases and one experimentally validated target database
2. Correlation analysis between miRNA and its predicted target genes on mRNA and protein expression levels derived from TCGA pancreatic adenocarcinoma (PAAD) project
3. Functional enrichment of significantly correlated miRNA targets

The novelty of our pipeline is the combination of including mature miRNA expression levels (isoform quantification) from TCGA-PAAD, protein expression levels from the cancer proteome atlas (TCPA) [24], and functional enrichment of both negatively and positively correlated miRNA-targets. Combination of the above-mentioned features in one tool may facilitate the research in miRNA biomarker discovery in pancreatic cancer. The tool was built in R and to make it even more accessible to users not familiar with R, we developed a shiny web app available at https://emmbor.shinyapps.io/mirfa/, where results for all miRNAs detected in TCGA-PAAD can be retrieved [17].

## Results

**Fig. 1:**
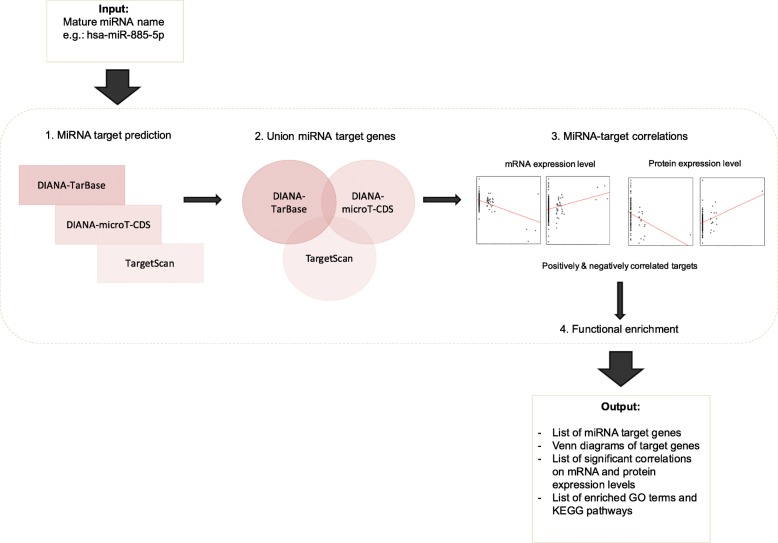
Overview of miRFA pipeline. The input is a mature miRNA name. MiRNA target prediction is performed in DIANA-Tarbase v7, DIANA-microT-CDS and TargetScan v7.1 (1.). The union of predicted miRNA targets (2.) were established as well as correlation values for miRNA-mRNA and miRNA-protein expression (3.). The list of correlated miRNA targets was subjected to functional enrichment analysis (4.) for gene ontology (GO) terms and Kyoto encyclopedia of genes and genomes (KEGG) pathways. The output is a list of miRNA target genes, Venn diagrams of target genes, significantly correlated target genes on mRNA and protein expression levels, and enriched GO terms and KEGG pathways

**Fig. 2:**
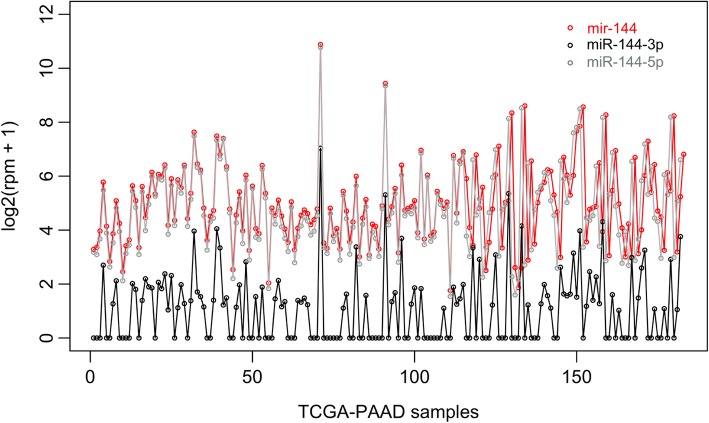
The difference between hsa-mir-144, hsa-miR-144-3p and hsa-miR-144-5p. Expression values were plotted for 183 TCGA-PAAD samples. Hsa-mir-144 (mir-144) represents the precursor hairpin expression, whereas hsa-miR-144-3p (miR-144-3p) and hsa-miR-144-5p (miR-144-5p) represents the mature miRNA isoforms expression. Rpm = reads per million counts, TCGA = the cancer genome atlas, PAAD = pancreatic adenocarcinoma

An overview of the miRFA pipeline is shown (Fig. 1). The input is a mature miRNA name and the output contains lists of miRNA target genes, Venn diagrams of target genes, miRNA targets correlations on mRNA and protein expression levels, and significantly enriched GO terms and KEGG pathways. For correlation analysis, we implemented miRNA isoform quantification data from TCGA in order to separate between expression levels of -3p and -5p arms of mature miRNAs. To illustrate the difference between expression levels of the precursor miRNA gene and the mature miRNA isoforms, hsa-mir-144 was plotted as an example together with expression levels of mature isoforms hsa-miR-144-3p and hsa-miR-144-5p (Fig. 2). The expression levels of the precursor hairpin hsa-mir-144 is more similar to the mature miRNA hsa-miR-144-5p compared to hsa-miR-144-3p.

### Predicted miRNA targets partially overlap

**Fig. 3:**
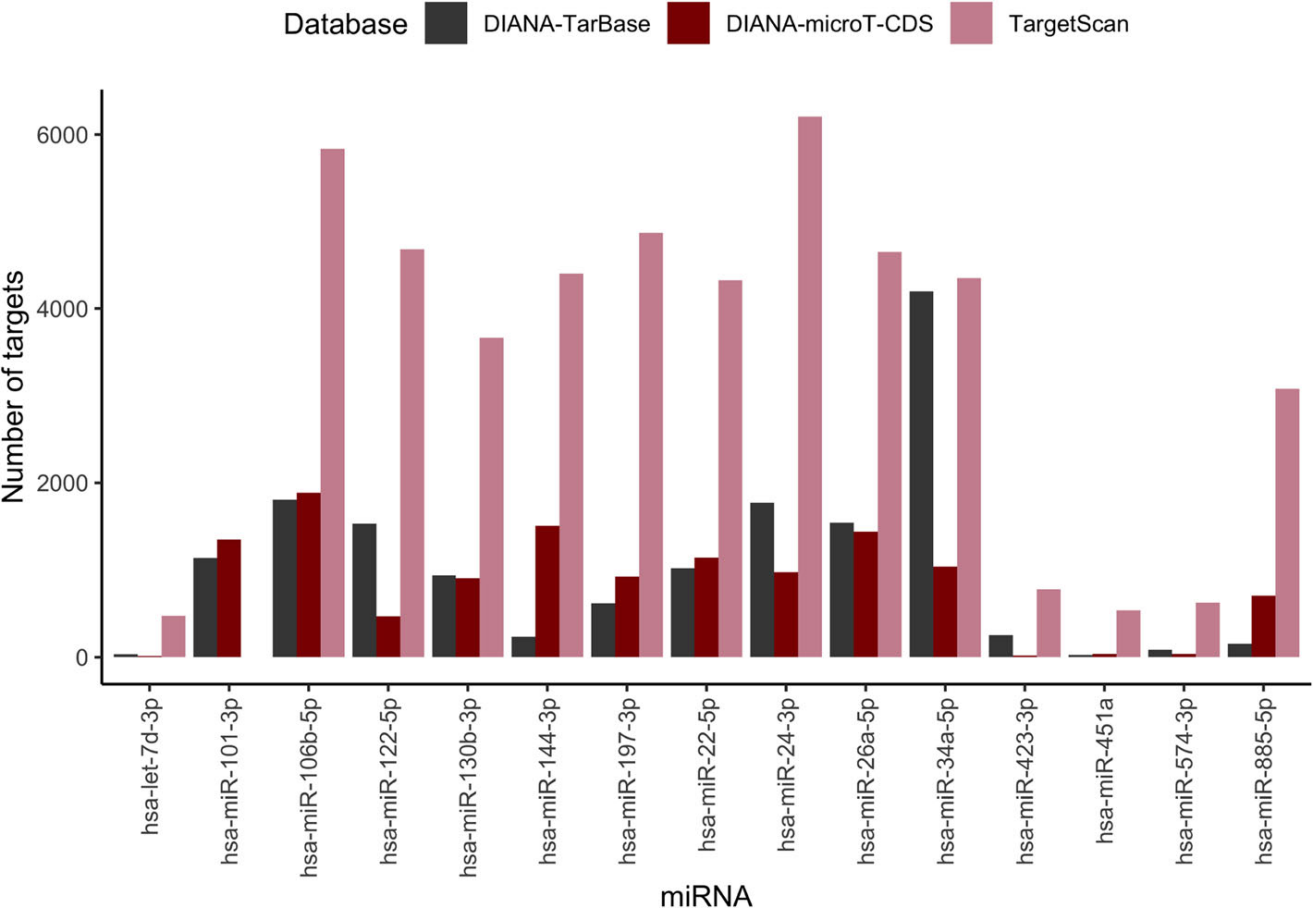
Number of predicted miRNA targets by DIANA-TarBase v7, DIANA-microT-CDS and TargetScan v7.1 for 15 miRNAs. The x axis shows every miRNA queried and the y axis shows the number of predicted miRNA targets

MiRNA target prediction was performed in three databases; DIANA-TarBase v7 [25], DIANA-microT-CDS [26] and TargetScan v7.1 [27]. The largest number of predicted targets was generally identified from TargetScan, exceeding 3000 predicted target genes for many of the miRNAs (Fig. 3). That said, no target gene was found in TargetScan for hsa-miR-101-3p.

A moderately sensitive threshold of 0.7 was used for DIANA-microT-CDS which affects the number of predicted miRNA targets. Defining a less restrictive threshold could generate more targets that are also present in DIANA-TarBase, but it could also introduce a higher number of false positives. The generated Venn diagrams show that some of the miRNA targets in DIANA-TarBase were not identified by the in silico prediction tools (Additional file 6: Figure S1). The opposite scenario also occurs, that targets predicted by TargetScan or DIANA-microT-CDS have not been experimentally validated.

### MiRNA-mRNA correlations

As miRNA target prediction tools can render many false positives, in silico evaluation data is useful to narrow down interesting gene candidates. To identify target genes that may have a role in pancreatic cancer progression, expression data of miRNAs, mRNAs, and proteins from pancreatic cancer tissue was used to analyze correlations between the query miRNA and its corresponding target genes on mRNA and protein levels.

**Fig. 4:**
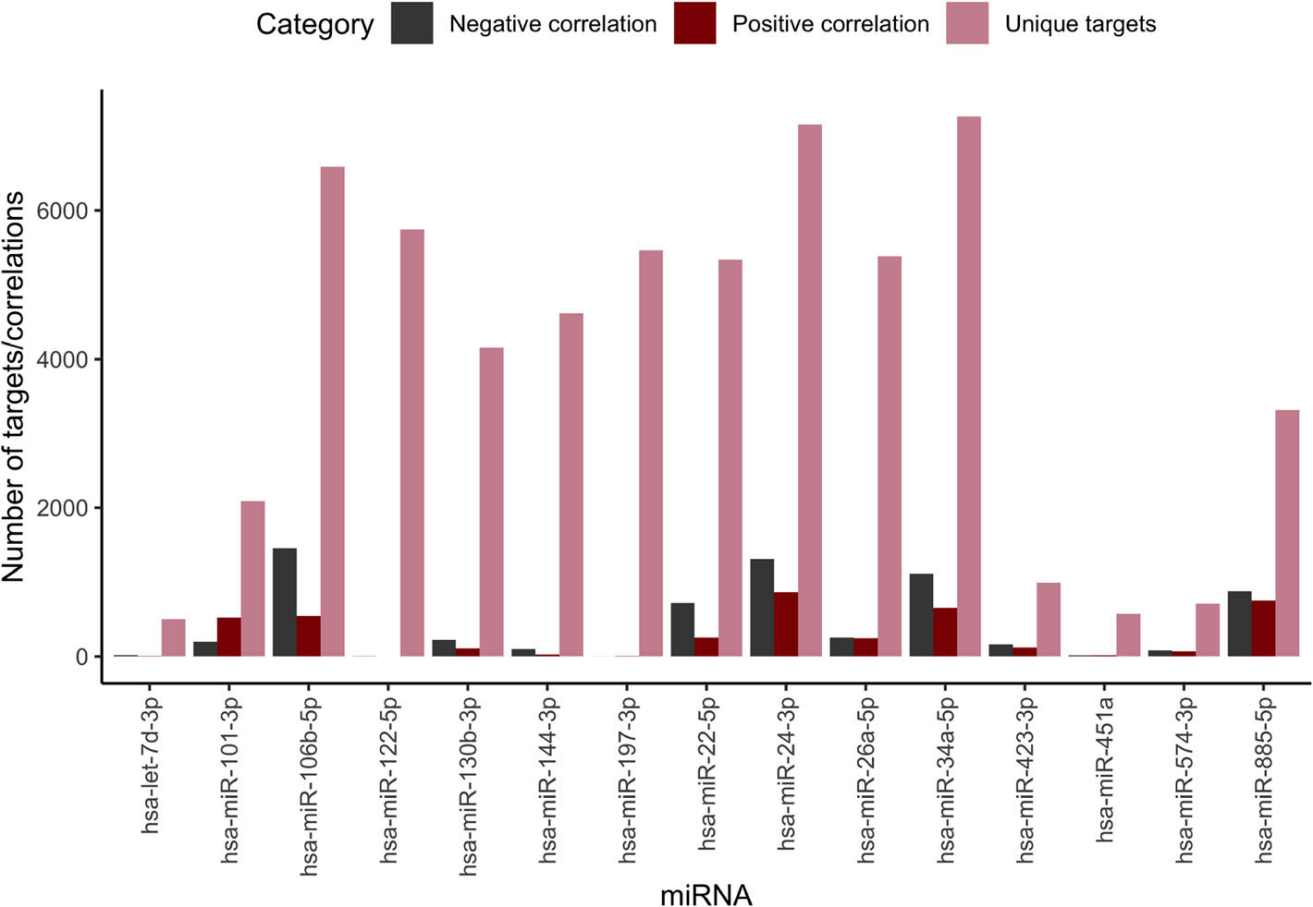
Number of predicted miRNA targets, positively correlated and negatively correlated miRNA targets on mRNA level (adjusted p-value < 0.05). The x axis shows each miRNA and the y axis shows number of genes (predicted miRNA targets or number of correlated genes). 'Unique targets' indicate the number of miRNA targets from the union of all three miRNA target prediction databases

In general, the number of significant correlations was low compared to the number of predicted targets (Fig. 4). For all 15 miRNAs combined, a total of 10,754 significant correlations (adjusted *p*-value < 0.05) were found, of which 4203 were positively correlated (Pearson’s correlation coefficient; PCC > 0), and 6551 negatively correlated (PCC <  0). Hsa-miR-106b-5p obtained the highest number of negative correlations and hsa-miR-24-3p the highest number of positive correlations.

### MiRNA-protein correlation

Correlation analysis of miRNA-protein expression levels was performed on 98 TCGA-PAAD samples. In total, 43 significant correlations (adjusted *p*-value < 0.05) were identified on protein level, consisting of 22 negatively correlated (PCC <  0) and 21 positively correlated (PCC > 0). Only five miRNAs (hsa-miR-24-3p, hsa-miR-885-5p, hsa-miR-101-3p, hsa-miR-34a-5p and hsa-miR-22-5p) were significantly correlated to any of its predicted miRNA targets on protein expression level (Table 1). Some genes, such as ‘FYN’, occurs more than once and the reason for this is that different antibodies have been used in reverse-phase protein arrays (RPPA) assay [24], and thus there will be multiple correlations for some miRNA-target pairs.

**Table 1:** Significant correlations between miRNA and its target gene on protein level

miRNA	Protein	PCC	Adjusted *P*-value
hsa-miR-101-3p	SRSF1	−0.32	0.023
hsa-miR-101-3p	FYN	0.31	0.023
hsa-miR-101-3p	FYN	0.28	0.048
hsa-miR-101-3p	PDCD4	0.31	0.023
hsa-miR-101-3p	MTOR	0.33	0.023
hsa-miR-885-5p	EGFR	−0.29	0.035
hsa-miR-885-5p	RAD51	−0.32	0.015
hsa-miR-885-5p	CDKN1B	0.34	0.015
hsa-miR-885-5p	MSH2	0.33	0.015
hsa-miR-885-5p	GSK3B	0.38	0.007
hsa-miR-885-5p	PRKAA1	0.33	0.015
hsa-miR-34a-5p	NDRG1	−0.33	0.035
hsa-miR-34a-5p	EIF4EBP1	−0.32	0.037
hsa-miR-34a-5p	EIF4EBP1	−0.35	0.026
hsa-miR-34a-5p	AKT1S1	−0.36	0.026
hsa-miR-24-3p	ASNS	−0.31	0.021
hsa-miR-24-3p	JAK2	−0.34	0.014
hsa-miR-24-3p	ATM	−0.30	0.024
hsa-miR-24-3p	CDK1	−0.36	0.014
hsa-miR-24-3p	YBX1	−0.31	0.021
hsa-miR-24-3p	EIF4EBP1	−0.31	0.021
hsa-miR-24-3p	FOXO3	−0.28	0.042
hsa-miR-24-3p	RPS6	−0.32	0.021
hsa-miR-24-3p	INPP4B	0.35	0.014
hsa-miR-24-3p	KDR	0.32	0.021
hsa-miR-24-3p	CCNB1	0.35	0.014
hsa-miR-24-3p	EGFR	0.34	0.014
hsa-miR-24-3p	IRS1	0.28	0.042
hsa-miR-24-3p	ITGA2	0.41	0.004
hsa-miR-24-3p	CASP3	0.31	0.021
hsa-miR-24-3p	YWHAZ	0.31	0.021
hsa-miR-22-5p	FYN	−0.28	0.049
hsa-miR-22-5p	SRC	−0.28	0.049
hsa-miR-22-5p	YBX1	−0.36	0.007
hsa-miR-22-5p	YES1	−0.28	0.049
hsa-miR-22-5p	LYN	−0.28	0.049
hsa-miR-22-5p	PTPN11	−0.36	0.007
hsa-miR-22-5p	MYH9	0.28	0.049
hsa-miR-22-5p	PEA15	0.37	0.007
hsa-miR-22-5p	CASP3	0.37	0.007
hsa-miR-22-5p	PIK3CA	0.29	0.049
hsa-miR-122-5p	MAPK14	−0.33	0.042
hsa-miR-122-5p	EIF4EBP1	0.52	0.000004

*PCC* Pearson’s correlation coefficient

### MiRNA-mRNA-protein integration

Sixteen miRNA-target gene pairs were significantly correlated at both mRNA and protein expression levels (Table 2). In 12 out of 16 correlations, the Pearson’s correlation coefficient had similar direction on mRNA and protein levels. For correlation between hsa-miR-24-3p – CDK1, the correlation is positive on mRNA expression level (PCC = 0.35) and negative on protein expression level (PCC = − 0.36). The opposite is observed for the correlated pairs hsa-miR-885-5p – PRKAA1, hsa-miR-24-3p – KDR and hsa-miR-22-5p – PIK3CA.

**Table 2:** Significant correlations on mRNA and protein expression levels

miRNA	Gene	PCC (mRNA level)	PCC (protein level)
hsa-miR-101-3p	FYN	0.33	0.31
hsa-miR-101-3p	FYN	0.33	0.28
hsa-miR-101-3p	PDCD4	0.40	0.31
hsa-miR-885-5p	CDKN1B	0.25	0.34
hsa-miR-885-5p	EGFR	−0.46	−0.29
hsa-miR-885-5p	PRKAA1	−0.28	0.33
hsa-miR-885-5p	RAD51	−0.47	−0.32
hsa-miR-24-3p	CCNB1	0.33	0.35
hsa-miR-24-3p	CDK1	0.35	−0.36
hsa-miR-24-3p	INPP4B	0.37	0.35
hsa-miR-24-3p	ITGA2	0.34	0.41
hsa-miR-24-3p	KDR	−0.24	0.32
hsa-miR-24-3p	RPS6	−0.25	−0.32
hsa-miR-24-3p	YWHAZ	0.51	0.31
hsa-miR-22-5p	PIK3CA	−0.20	0.29
hsa-miR-22-5p	PTPN11	−0.21	−0.36

*PCC* Pearson’s correlation coefficient

### Functional enrichment analysis

Predicted miRNA targets that have been filtered out as more reliable due to correlation with corresponding miRNAs were evaluated further by performing functional enrichment analysis. The most commonly occurring top GO term for all miRNA targets combined was binding (GO:0005488) or protein binding (GO:0005515) for molecular function (Table 3), and for biological process, no specific GO term was overrepresented among the 15 miRNAs studied (Table 4). For cellular compartment (Table 5), 6 miRNAs had a top GO term connected to intracellular parts (GO:0005622 and GO:0044424). Two miRNAs (hsa-miR-34a-5p and hsa-miR-885-5p) associated to pancreas-related GO terms. Hsa-miR-34a-5p was associated to GO:0031018; endocrine pancreas development and hsa-miR-885-5p to GO:0003309; type B pancreatic cell differentiation. The miRNAs that did not have any enriched targets for GO terms or KEGG pathways were excluded from Tables 3, 4, 5 and 6.

**Table 3:** Top significant molecular function GO term for each miRNA. ‘Count’ represents number of miRNA targets enriched

miRNA	GO term	Count	*P*-value
hsa-let-7d-3p	GO:0140110 transcription regulator activity	8	0.004
hsa-miR-101-3p	GO:0005488 binding	622	< 0.001
hsa-miR-106b-5p	GO:0005488 binding	1633	< 0.001
hsa-miR-130b-3p	GO:0005024 transforming growth factor beta-activated receptor activity	5	< 0.001
hsa-miR-144-3p	GO:0005515 protein binding	91	< 0.001
hsa-miR-197-3p	GO:1901363 heterocyclic compound binding	5	0.011
hsa-miR-22-5p	GO:0005515 protein binding	695	< 0.001
hsa-miR-24-3p	GO:0005515 protein binding	1447	< 0.001
hsa-miR-26a-5p	GO:0005488 binding	423	< 0.001
hsa-miR-34a-5p	GO:0005488 binding	1442	< 0.001
hsa-miR-423-3p	GO:0044212 transcription regulatory region DNA binding	31	< 0.001
hsa-miR-451a	GO:0005515 protein binding	23	0.011
hsa-miR-574-3p	GO:0003674 molecular_function	147	0.001
hsa-miR-885-5p	GO:0005488 binding	1335	< 0.001

*NA* not applicable

**Table 4:** Top significant biological process GO term for each miRNA. ‘Count’ represents number of miRNA targets enriched

miRNA	GO term	Count	*P*-value
hsa-let-7d-3p	GO:0051962 positive regulation of nervous system development	5	0.001
hsa-miR-101-3p	GO:0019219 regulation of nucleobase-containing compound metabolic process	249	< 0.001
hsa-miR-106b-5p	GO:0007399 nervous system development	356	< 0.001
hsa-miR-130b-3p	GO:0046834 lipid phosphorylation	11	< 0.001
hsa-miR-144-3p	GO:0072659 protein localization to plasma membrane	7	0.001
hsa-miR-22-5p	GO:0051641 cellular localization	253	< 0.001
hsa-miR-24-3p	GO:0023051 regulation of signaling	516	< 0.001
hsa-miR-26a-5p	GO:0002009 morphogenesis of an epithelium	28	< 0.001
hsa-miR-34a-5p	GO:0050794 regulation of cellular process	1084	< 0.001
hsa-miR-423-3p	GO:0065009 regulation of molecular function	77	< 0.001
hsa-miR-451a	GO:0000165 MAPK cascade	8	< 0.001
hsa-miR-574-3p	GO:0071495 cellular response to endogenous stimulus	22	0.001
hsa-miR-885-5p	GO:0031323 regulation of cellular metabolic process	603	< 0.001

*NA* not applicable

**Table 5:** Top significant cellular component GO term for each miRNA. ‘Count’ represents number of miRNA targets enriched

miRNA	GO term	Count	*P*-value
hsa-let-7d-3p	GO:0044459 plasma membrane part	8	0.026
hsa-miR-101-3p	GO:0005654 nucleoplasm	224	< 0.001
hsa-miR-106b-5p	GO:0005622 intracellular	1630	< 0.001
hsa-miR-130b-3p	GO:0044444 cytoplasmic part	196	< 0.001
hsa-miR-144-3p	GO:0070161 anchoring junction	14	< 0.001
hsa-miR-22-5p	GO:0044424 intracellular part	828	< 0.001
hsa-miR-24-3p	GO:0005737 cytoplasm	1388	< 0.001
hsa-miR-26a-5p	GO:0044424 intracellular part	427	< 0.001
hsa-miR-34a-5p	GO:0005622 intracellular	1431	< 0.001
hsa-miR-423-3p	GO:0005737 cytoplasm	191	< 0.001
hsa-miR-451a	GO:0031252 cell leading edge	6	< 0.001
hsa-miR-574-3p	GO:0044424 intracellular part	132	< 0.001
hsa-miR-885-5p	GO:0005622 intracellular	1322	< 0.001

*NA* not applicable

**Table 6:** Top significant KEGG pathway for each miRNA. ‘Count’ represents number of miRNA targets enriched

miRNA	Pathway	Count	*P*-value
hsa-miR-101-3p	path:hsa04070 Phosphatidylinositol signaling system	13	< 0.001
hsa-miR-106b-5p	path:hsa04015 Rap1 signaling pathway	46	< 0.001
hsa-miR-130b-3p	path:hsa04350 TGF-beta signaling pathway	8	< 0.001
hsa-miR-144-3p	path:hsa05200 Pathways in cancer	7	0.041
hsa-miR-22-5p	path:hsa04360 Axon guidance	23	< 0.001
hsa-miR-24-3p	path:hsa05100 Bacterial invasion of epithelial cells	24	< 0.001
hsa-miR-26a-5p	path:hsa04510 Focal adhesion	15	< 0.001
hsa-miR-34a-5p	path:hsa04810 Regulation of actin cytoskeleton	38	< 0.001
hsa-miR-423-3p	path:hsa04015 Rap1 signaling pathway	13	< 0.001
hsa-miR-574-3p	path:hsa01522 Endocrine resistance	6	< 0.001
hsa-miR-885-5p	path:hsa05160 Hepatitis C	28	< 0.001

*NA* not applicable

The top KEGG pathway varied among the miRNAs but the Rap1 signaling pathway (path:hsa04015) was the only one that occurred more than once (Table 6). No GO term or KEGG pathway enrichment was found for correlated miRNA targets of hsa-let-7d-3p, hsa-miR-122-5p, hsa-miR-197-3p or hsa-miR-451a.

### Overlap of miRNAs

Initially, we were interested to see if there are any shared targets between our panel of 15 differentially expressed miRNAs. No overlap of predicted miRNA targets was detected for all 15 miRNAs combined. However, by studying the established list of their enriched KEGG pathways, we could determine four miRNAs (hsa-miR-22-5p, hsa-miR-24-3p, hsa-miR-106b-5p and hsa-miR-885-5p) associated to hsa0512 ‘Pancreatic cancer’ (see Additional files 1, 2, 3 and 4). Based on this finding, miRNA target genes shared between these four miRNAs were studied further. Sixteen overlapping significantly correlated miRNA target genes were identified (Table 7). Nuclear factor I B (NFIB) shows similar correlation coefficients between these four miRNAs.

**Table 7:** Pearson’s correlation coefficient shown for overlapping predicted miRNA target genes of four miRNAs

Gene	hsa-miR-22-5p	hsa-miR-24-3p	hsa-miR-106b-5p	hsa-miR-885-5p
MAP 1B	- 0.24	- 0.20	- 0.40	0.33
NFIB	- 0.19	- 0.33	- 0.35	- 0.29
REV3L	- 0.22	- 0.28	- 0.30	0.18
LONRF2	- 0.24	- 0.26	- 0.34	0.34
TMTC1	- 0.24	- 0.22	- 0.21	0.21
MSANTD4	- 0.30	- 0.18	- 0.29	0.22
HCN1	- 0.23	- 0.24	- 0.33	0.17
SIK2	- 0.23	- 0.21	- 0.22	0.38
GABRG2	- 0.22	- 0.19	- 0.24	0.33
DCX	- 0.20	- 0.21	- 0.22	0.25
KCND3	- 0.21	- 0.28	- 0.35	0.28
NTRK2	- 0.19	- 0.28	- 0.23	0.19
CNTNAP5	- 0.21	- 0.24	- 0.28	0.21
FRRS1L	- 0.32	- 0.22	- 0.36	0.37
MGAT4C	- 0.21	- 0.31	- 0.28	0.44
TMEM134	0.32	0.18	0.27	- 0.19

### Survival analysis

**Fig. 5:**
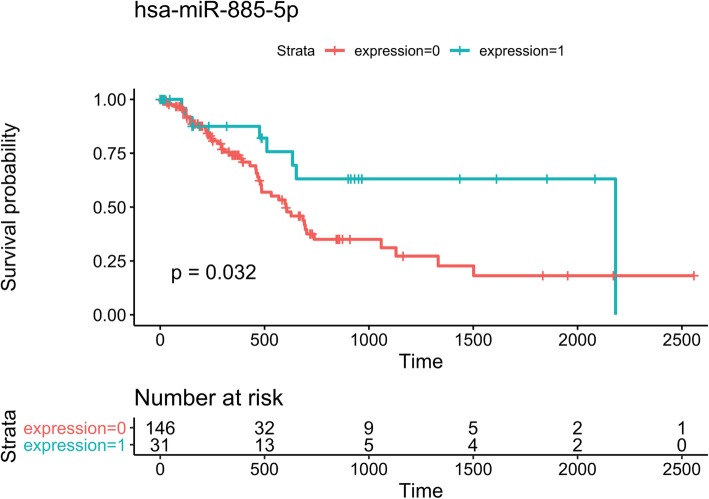
Overall survival for hsa-miR-885-5p using median log2(rpm + 1) expression as cut-off. Expression = 0 is the group that has a value below median and expression = 1 is the group that has a value above median. The nominal *p*-value is displayed (*p* = 0.032), but was not significant after multiple hypothesis correction using Benjamini-Hochberg

Due to many identified correlations observed between the miRNAs and their target genes suggesting a regulatory role in pancreatic cancer, we further studied the fifteen miRNAs as prognostic biomarkers by Kaplan-Meier survival analysis. The median was used as cut-off and hsa-miR-885-5p was found to be significantly correlated to survival (Fig. 5, nominal *p*-value = 0.032). However, after adjusting for multiple hypothesis testing, none of the 15 miRNAs analyzed was significant for overall survival in the TCGA-PAAD dataset (Additional file 6: Figure S2).

### Network analysis of hsa-miR-885-5p targets

**Fig. 6:**
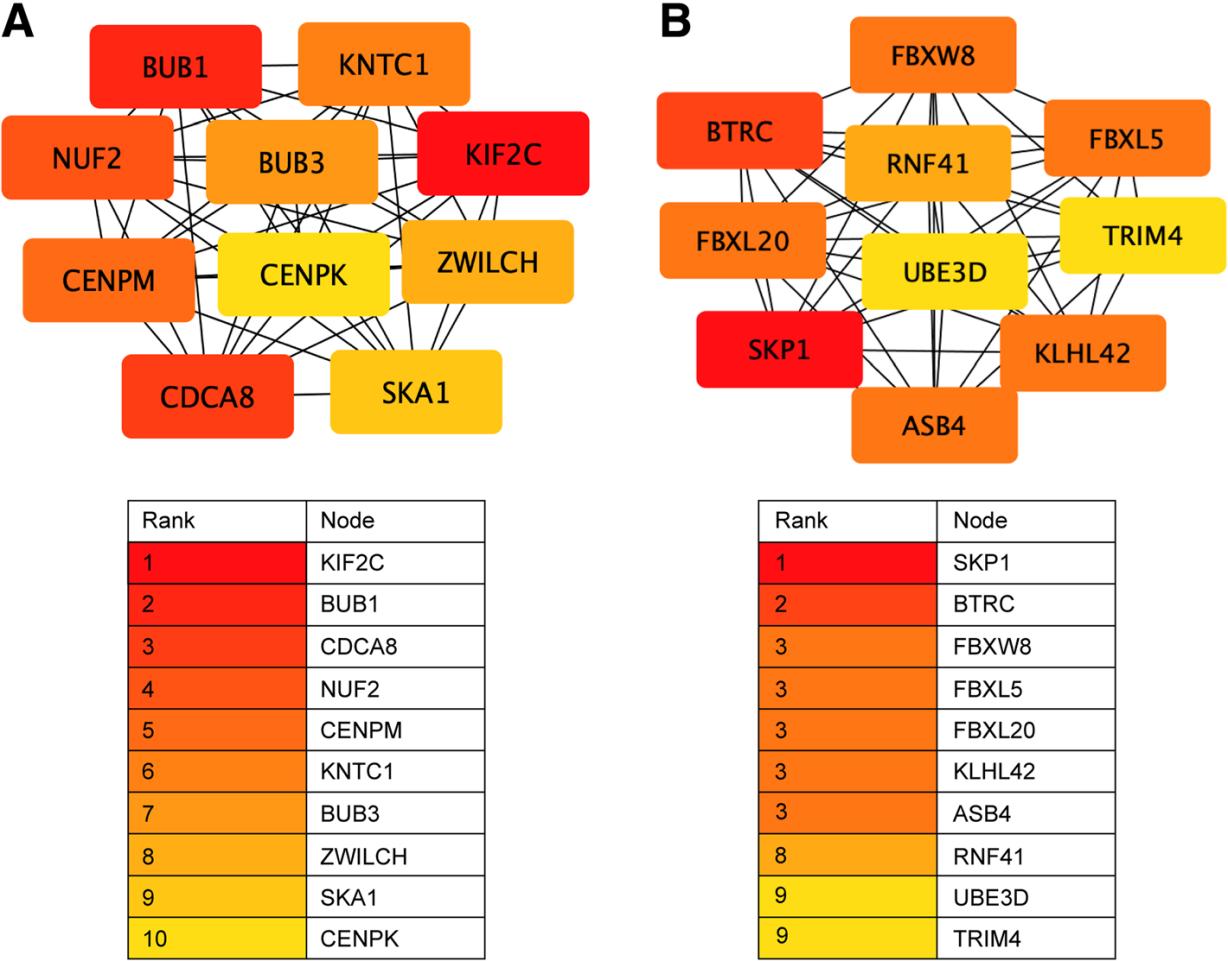
Hub genes for hsa-miR-885-5p. Top 10 hub genes and their ranks are shown for negatively correlated (**a**) and positively correlated (**b**) targets

**Fig. 7:**
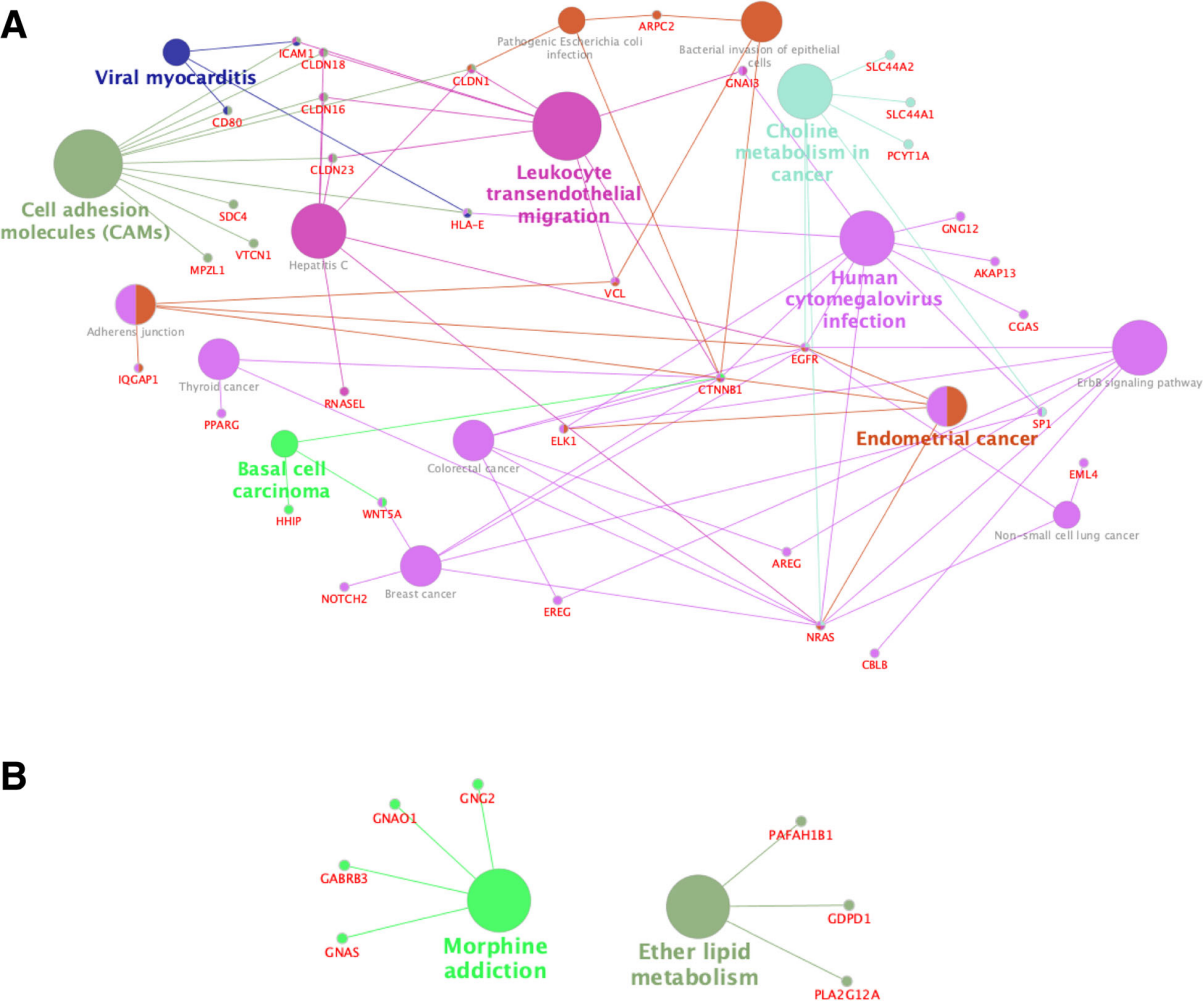
Enriched KEGG pathways generated by ClueGO and Cluepedia. Networks were generated separately for (**a**) negatively correlated (PCC ≤ −0.4) and (**b**) positively correlated (PCC > 0.4) targets. The size of the nodes corresponds to the significance of the KEGG pathway

The correlated miRNA target genes can be used for other downstream analyses, one example used here is network analyses. For this, we used hsa-miR-885-5p as an example and analyzed negatively and positively correlated targets separately. Hub genes were extracted (Fig. 6), where the top 10 connected proteins are shown together with the rank of each hub gene. ClueGO and CluePedia were used to visualize the interplay between significant KEGG pathways and to see which genes connect the pathways (Fig. 7). Negatively and positively correlated gene targets were handled separately. To narrow down the number of targets analyzed, a correlation coefficient cut-off of 0.4 (positive correlations) or − 0.4 (negative correlations) was used. Consequently, only target genes correlating on mRNA expression levels were included in these analyses as the targets correlated on protein expression levels were below this cutoff. Three genes are shared between many pathways in the negatively correlated network (Fig. 7a); EGFR (9 pathways), CTNNB1 (10 pathways) and NRAS (9 pathways).

### Comparison to other tools

MiRFA has the strength of combining miRNA target prediction and correlation analyses (positive and negative correlations) on both mRNA and protein expression levels. Furthermore, miRFA includes mature miRNA expression in the correlation analyses and performs functional enrichment of the correlated targets. Another strength of our tool is that it is also web-based. We compared our tools to others that perform miRNA functional analysis or functional annotations (Table 8). MiRFA and miRCancerdb [16] are both available as R packages and web-based tools. MultiMiR [12], RBiomirGS [13], MiRComb [14] and miRLab [15] are only available as R packages, whereas MiRpath [18], miEAA [28], TAM [29] and GeneTrail2 [30] are web-based resources. Four tools (miRFA, miRCancerdb, miRComb, miRLab) take into account correlation analysis in combination with miRNA target prediction. Our tool does not provide information on miRNA annotation such as miRNA clusters or families that can be obtained using miEAA or TAM tools. Furthermore, our tool does not offer a functional analysis of precursor hairpin miRNAs and is restricted to pancreatic cancer in its current form.

**Table 8:** Comparison of miRFA to other available tools for functional analysis of miRNAs

Feature		Tool									
		miRFA	miRCancerdb	multiMiR	RBiomirGS	MiRComb	miRLab	miRpath	miEAA	TAM	GeneTrail2
Platform		R/Web-based	R/Web-based	R	R	R	R	Web-based	Web-based	Web-based	Web-based
MiRNA input/expression	Mature miRNA	✓		✓	✓	✓	^a^	✓	✓		✓
	Precursor miRNA		✓			✓	✓		✓	✓	
MiRNA target prediction database	Experimentally validated database(s)	✓		✓	✓		✓	✓	✓		✓
	Target prediction database(s)	✓	✓	✓	✓	✓	✓	✓			
Correlation analysis expression levels	mRNA expression	✓	✓			✓	✓				
	Protein expression	✓	✓								
	Negative correlation	✓	✓			✓	✓^b^				
	Positive correlation	✓	✓								
Functional enrichment		✓			✓	✓	✓	✓	✓	✓	✓
Disease-specific		✓	✓	✓			✓				
miRNA annotation ^c^									✓	✓	✓

^a^ Based on if the user implements built in TCGA retriever

^b^ Negative correlations are ranked at the top

^c^ Includes cluster, family, functions, diseases, tissue specificity

In addition to the feature comparison between tools, shown in Table 8, we analyzed our list of 15 miRNAs (Tables 9 and 10) in miRCancerdb. Since MiRCancerdb does not provide the option to analyze functional enrichment, this feature was not considered for a comparison. In order to obtain all correlated targets in miRCancerdb, we set a threshold to 10,000 correlations, and select parameters ‘PAAD’ for TCGA study code, ‘Targets only’ for feature type and both direction of correlation with an absolute minimum of 0 for correlation. MiRCancerdb has filtered out correlations less than 0.1 so these correlations were not included in our comparison since we used the web-based tool [16]. Since miRCancerdb is built with precursor miRNAs, we used the precursor names of our 15 miRNAs. To benchmark miRCancerdb with our tool, we used the genes list from KEGG pathway hsa05212 pancreatic cancer (75 genes) and counted how many pancreatic cancer-related genes were obtained in the two tools (Tables 9 and 10). MiRNAs with 0 correlated targets in both tools were excluded from the tables. MiRCancerdb generates some overlap of correlated targets between has-mir-144 (miRCancerdb) and hsa-miR-144-3p (miRFA), but we can also find overlap of correlated targets between mir-144 (miRCancerdb) and the other mature miRNA; hsa-miR-144-5p (miRFA).

**Table 9:** Comparison of correlated targets between miRCancerdb and miRFA on mRNA level. KEGG genes refer to genes included in KEGG pathway pancreatic cancer

miRCancerdb				miRFA			
Input	Cor mRNA	KEGG genes	Percentage (%)	Input	Cor mRNA	KEGG genes	Percentage (%)
hsa-mir-144	284	1	0.4	hsa-miR-144-3p	127	19	15.0
hsa-mir-106b	458	6	1.3	hsa-miR-106b-5p	2005	14	0.7
hsa-mir-451a	0	NA	NA	hsa-miR-451a	29	0	0
hsa-mir-101	0	NA	NA	hsa-miR-101-3p	713	5	0.7
hsa-mir-26a	0	NA	NA	hsa-miR-26a-5p	508	0	0
hsa-mir-574	0	NA	NA	hsa-miR-574-3p	156	2	1.3
hsa-mir-885	0	NA	NA	hsa-miR-885-5p	1642	12	0.7
hsa-mir-130b	277	3	1.1	hsa-miR-130b-3p	333	3	0.9
hsa-mir-34a	176	2	1.1	hsa-miR-34a-5p	1774	10	0.6
hsa-mir-24	0	NA	NA	hsa-miR-24-3p	2179	15	0.7
hsa-mir-22	261	4	1.5	hsa-miR-22-5p	977	8	0.8
hsa-let-7d	443	5	1.1	hsa-let-7d-3p	28	0	0
hsa-mir-197	57	0	0	hsa-miR-197-3p	7	0	0
hsa-mir-423	0	NA	NA	hsa-miR-423-3p	288	2	0.7
hsa-mir-122	50	0	0	hsa-miR-122-5p	3	0	0

Cor mRNA = number of correlated miRNA – targets on mRNA level, KEGG genes = number of genes associated to KEGG pathway hsa05212 pancreatic cancer, percentage = percentage of KEGG genes compared to total number of correlated targets, NA = not available

**Table 10:** Comparison of correlated targets between miRCancerdb and miRFA on protein expression level

miRCancerdb					miRFA				
Input	Cor protein	KEGG genes	Percentage (%)	Gene names	Input	Cor protein	KEGG genes	Percentage (%)	Gene names
hsa-mir-144	5	0	0	NA	hsa-miR-144-3p	0	NA	NA	NA
hsa-mir-106b	3	2	67	AKT1/2/3, RB1	hsa-miR-106b-5p	0	NA	NA	NA
hsa-mir-101	0	NA	NA	NA	hsa-miR-101-3p	5	1	20	MTOR
hsa-mir-885	0	NA	NA	NA	hsa-miR-885-5p	6	2	33	EGFR, RAD51
hsa-mir-130b	3	1	33	SMAD4	hsa-miR-130b-3p	0	NA	NA	NA
hsa-mir-34a	2	1	50	SMAD4	hsa-miR-34a-5p	4	0	0	NA
hsa-mir-24	0	NA	NA	NA	hsa-miR-24-3p	16	1	6	EGFR
hsa-mir-22	3	2	67	AKT1/2/3, CDKN1A	hsa-miR-22-5p	10	1	10	PIK3CA
hsa-let-7d	4	3	75	AKT1/2/3, CDKN1A, RB1	hsa-let-7d-3p	0	NA	NA	NA
hsa-mir-122	0	NA	NA	NA	hsa-miR-122-5p	2	0	0	NA

Cor protein = number of correlated miRNA – targets on mRNA level, KEGG genes = number of genes associated to KEGG pathway hsa05212 pancreatic cancer, percentage = percentage of KEGG genes compared to total number of correlated targets, NA = not available

## Discussion

The aim of this study was to build a bioinformatics pipeline for miRNA functional analysis and correlation analyses for in silico evaluation (Fig. 1). Expression data of mature miRNA isoforms was included in correlation analyses since the differentially expressed mature miRNAs were used as input miRNAs in the pipeline (Fig. 2). Many of the TCGA samples showed expression in hsa-miR-144-3p and not in hsa-miR-144-5p. Relying on the precursor hsa-mir-144 expression would have caused false-positive expression values as the precursor hsa-mir-144 expression pattern is more similar to the expression of the -5p mature miRNA in this case. The pipeline generated miRNA targets, correlated targets, enriched GO terms and KEGG pathways for 15 miRNAs. This study utilized input miRNAs detected in plasma samples of PDAC patients [7], whereas the expression data used for correlation analyses originated from tumor tissue. The circulating miRNAs could be a leakage from the tumor or a systemic response to the cancer state.

MiRNA target prediction tends to generate a lot of false-positives [19], which is why correlation analyses between each miRNA and its predicted targets were performed as an in silico evaluation. Correlation analysis is one way of determining the dependency between two variables [31] and was applied on expression levels of miRNA and its target genes on both mRNA and protein levels in this study. Correlation analyses do not automatically indicate that the dependency is direct, however, since the miRNA-gene pairs were predicted to interact, it gives a stronger support for a miRNA-mediated regulation effect. Including the correlation analyses saves time in post-processing steps of extracting interesting miRNA target candidates since the output list of interesting candidates becomes shorter after in silico evaluation.

The number of correlated miRNA-target pairs (on mRNA expression level) were not associated to the number of targets predicted by the databases (Fig. 4), i.e. that a higher number of predicted miRNA targets would automatically generate a higher number of significant correlations. In the study by Seo et al. [21], protein expression data was included in the correlations as miRNA-mediated regulation acts post-transcriptionally and thus mainly affects the protein expression levels. MiRNAs regulate their targets by degradation or repression and an effect on the protein level might not always be visible on mRNA level [4]. Hence, when possible, the protein expression levels are useful in correlation-based in silico evaluation. One limitation for using correlation analyses based on mRNA and protein expression data is the risk for false negatives, due to missing expression data for some predicted targets, especially for the protein expression data in this case. TCPA provide expression data for around 200 proteins and resulted in only 43 significant correlations (Table 1) as compared to a total of 10,754 correlated miRNA-target pairs on mRNA expression level (see Additional file 5) accounting for all 15 miRNAs. Hence, there is a need for more high-throughput proteomics for miRNA functional analysis purposes. No feature was included in the pipeline to show which targets were not available among mRNA or protein expression data.

A possible drawback of our pipeline is introduction of false positive correlations between miRNAs and its targets. The trade-off between specificity and sensitivity in biomarker discovery is always of great importance. Our intention with the proposed pipeline is to provide a tool that will support an early phase of exploratory research on candidate biomarkers in heterogeneous diseases. Given that premise, we suggest that the value of finding novel important biomarkers may override the concern with introducing false connections.

Kaplan-Meier survival analysis suggests that hsa-miR-885-5p may act as a tumor suppressor in PDAC (Fig. 5). This is supported by previous functional studies of hsa-miR-885-5p. Hsa-miR-885-5p was previously identified to act as a tumor suppressor in hepatocellular carcinoma [32] and hepatoblastoma [33] by targeting β-catenin. Furthermore, a decay in expression correlated to a more progressed hepatocellular carcinoma by correlation to tumor-node-metastasis (TNM) stages [32]. In this study, β-catenin (CTNNB1) was predicted as a target for hsa-miR-885-5p by TargetScan v7.1 and DIANA-microT-CDS, and a significant negative correlation on mRNA level (PCC = − 0.46) was identified. In addition, hsa-miR-885-5p has previously been found up-regulated in liver metastases compared to the primary tumor in colorectal cancer [34], and a regulation involving its predicted target CPEB2 has been suggested [24]. CPEB2 was identified as a target for hsa-miR-885-5p by TargetScan and DIANA-microT-CDS in this study but was not significantly correlated. In addition, several studies support hsa-miR-885-5p as a circulating biomarker in PDAC [7, 9, 35, 36] .

MiRNA target prediction lacks information about other factors that could affect the extent of miRNA-mediated regulation. Hence, further validation is needed to increase the reliability of the identified targets and extract genes of interest in a disease-specific context, in this case pancreatic cancer. Experimental validation and additional bioinformatics analyses can be applied to correlated miRNA targets, such as functional enrichment (Tables 3, 4, 5 and 6) and network analyses (Figs. 6 and 7). Our network analysis resulted in the top ranked hub genes KIF2C (kinesin family member 2C, also known as MCAK) for negatively correlated targets (Fig. 6a) and SKP1 (S-phase kinase associated protein 1) for positively correlated targets (Fig. 6b). KIF2C is involved in mitosis by associating to the centromere [37]. BUB1 (BUB1 mitotic checkpoint serine/threonine kinase) was found as top 2 for the negatively correlated targets and is active in a complex together with BUB3 [38], which is also among the hub genes for negatively correlated targets (Fig. 6a). This complex (BUB1/BUBR1/BUB3) is necessary for correct balance of kinase-phosphatase balance during mitosis and has been proven to have a role in chromosome instability and tumor progression as well [38]. Skp1 has been suggested to have a role in various cancer forms by contributing to active oncogenic (Skp1)-Cullin1-F-box protein (SCF) complexes [39]. SCF complexes is the best characterized E3 ligases and are involved in protein degradation.

NRAS, EGFR and CTNNB1 (β-catenin) were found to overlap between many enriched KEGG pathways for hsa-miR-885-5p negatively correlated targets (Fig. 7a). Expression of these three genes in a PDAC context has been studied previously. Overexpression of EGFR is detected in a fraction of PDAC patients with a range of 30.4–64.2% in different PDAC cohorts  [40–45]. β-catenin expression in PDAC tissue was previously reported as a prognostic marker, where a high expression using immunohistochemistry staining predicted longer survival [46]. NRAS has also been suggested as a protective biomarker in PDAC as assessed by immunohistochemistry [47]. A high fraction of PDAC patients harbor KRAS mutations,

91% in the TCGA-PAAD dataset, whereas NRAS mutation are rare [47]. KRAS was identified as a miRNA target and significantly correlated (PCC = − 0.19) with hsa-miR-885-5p (see Additional file 5). NRAS showed stronger correlation to hsa-miR-885-5p (PCC = − 0.42) than KRAS.

As highlighted in the comparison between our tool and other miRNA functional analysis tools, many resources exist for this type of analysis (Table 8). Although many tools resemble each other in terms of provided features, they still have significant differences and make up a very broad toolkit to apply in miRNA functional analysis. Hence, we identified an important gap to fill by developing a tool for correlating both mRNA and protein expression levels using mature miRNA isoforms expression levels. The mechanisms behind miRNA-mediated regulation are highly complex and act in a disease- or tissue-specific manner [48].

MiRFA was compared to miRCancerdb with regards to our list of 15 miRNAs (Tables 9 and 10). It is difficult to assess the performances of these tools in terms of prediction accuracy, since we do not know true miRNA targets in pancreatic cancer. Still, to be able to perform an objective comparison, we selected a benchmarking dataset consisting of 75 genes found in KEGG pathway hsa05212 pancreatic cancer. We calculated the fraction of pancreatic cancer pathway-associated genes identified by each of the tool (miRCancerdb and miRFA, Tables 9 and 10). The main difference between these tools is that miRCancerdb implements the miRNA-seq data from TCGA-PAAD while miRFA implements pre-processed mature miRNA isoform quantification expression data. The correlation dataset available in miRCancerdb is restricted to correlations above 0.1, whereas we have applied a threshold of adjusted *p*-value < 0.05.

Correlations were not obtained for all miRNAs in miRCancerdb. The targetscan R package targetscan. Hs.eg.db [49] was used to obtain targetscan targets in miRCancerdb. This R package is restricted to prediction of conserved miRNA targets only, which could explain why we do not obtain any correlations for some miRNAs and why the number of correlated targets is much higher in our tool for some miRNAs. In our tool we implemented the database for non-conserved miRNA targets as well and we also implemented DIANA-Tarbase and DIANA-microT-CDS in the miRNA target prediction step. For the comparison on protein expression levels (Table 10), a few pancreatic cancer-associated proteins were found in both tools. Interestingly, each of the tools also identified a unique set of true targets from the defined benchmarking set of KEGG pathway pancreatic cancer, suggesting that it might be of interest to use both tools for studying miRNA functions in pancreatic cancer. In addition to genes in KEGG pathway pancreatic cancer, there could of course be other relevant targets to study in a pancreatic cancer context.

The limitation of our pipeline is its current restriction to pancreatic cancer, as the correlations are based on data derived from pancreatic cancer tissue. To expand the pipeline for other cancer types, the pre-processing and inclusion of the miRNA, mRNA and protein expression levels is required for each cancer type. We are currently extending the pipeline to provide the same functionality for breast cancer miRNAs.

## Conclusions

The developed pipeline is proven useful for generating shortlist of relevant target genes for 15 miRNAs that are differentially expressed in PDAC, along with their enriched GO terms, KEGG pathways, and significant correlations. Predicted miRNA-mRNA interactions in conjunction with correlation analyses of expression levels provides support for miRNA-mediated regulation. The pipeline is applicable to any mature miRNA in the context of pancreatic cancer. In the future, this pipeline could be further developed to include other cancer types by incorporating the corresponding miRNA, mRNA and protein expression levels of other TCGA cancer types. Our results and previously published data suggest that hsa-miR-885-5p could act as a tumor suppressor and should be experimentally validated in PDAC.

## Methods

### Data

#### Differentially expressed microRNAs

A published dataset of 15 significantly altered miRNAs detected in plasma of PDAC patients at the time of diagnosis were used in this study (Table 11) [7]. These circulating miRNAs have been identified in plasma samples from patients diagnosed with PDAC and admitted for surgery at the Department of Surgery, Umeå university hospital. MiRNA isolates from 23 PDAC patients and 22 controls were analyzed by RT-qPCR for 372 validated miRNAs using Human Panel I (V.4, Exiqon, Vedbaek, Denmark). The combination of these 15 miRNAs generated an AUC of 0.96 compared to 0.92 for CA 19–9 at time of diagnosis.

**Table 11:** Significantly altered plasma miRNAs in pancreatic cancer patients [7]. Regulation describes whether the miRNA was found up- or down-regulated in pancreatic cancer patients

miRNA	Regulation	FC
hsa-miR-144-3p	Down	0.4
hsa-miR-106b-5p	Down	0.8
hsa-miR-451a	Down	0.5
hsa-miR-101-3p	Down	0.7
hsa-miR-26a-5p	Down	0.6
hsa-miR-574-3p	Up	1.5
hsa-miR-885-5p	Up	3.9
hsa-miR-130b-3p	Up	1.5
hsa-miR-34a-5p	Up	2.2
hsa-miR-24-3p	Up	1.2
hsa-miR-22-5p	Up	1.4
hsa-let-7d-3p	Up	1.3
hsa-miR-197-3p	Up	1.4
hsa-miR-423-3p	Up	1.3
hsa-miR-122-5p	Up	2.5

*FC* fold change

#### Expression data

MiRNA and mRNA expression data have previously been generated by next-generation sequencing (seq) within TCGA Research Network (http://cancergenome.nih.gov/). The miRNA-seq isoform expression quantification data files available at the GDC portal (https://portal.gdc.cancer.gov/) from pancreatic adenocarcinoma (TCGA-PAAD) samples were used. For mRNA expression data, log2(fpkm + 1) values for 183 samples were downloaded from xena browser (https://xenabrowser.net/datapages/). TCGA-PAAD expression data were derived from 1 epithelial neoplasm, 5 cystic, mucinous or serous neoplasms, 31 adenomas or adenocarcinomas and 146 ductal or lobular neoplasms. The R package org. Hs.eg.db was used for converting Ensembl-IDs to HUGO Gene Nomenclature Committee (HGNC) symbols [50].

Protein expression levels of 218 proteins, analyzed by RPPA, on tissue samples provided by TCGA were obtained from 98 TCGA-PAAD samples [24]. Level 4 protein expression data was downloaded from TCPA (http://bioinformatics.mdanderson.org/main/TCPA:Overview) by accessing the data portal (http://tcpaportal.org/tcpa/download.html). TCPA expression data were derived from 2 cystic, mucinous or serous neoplasms, 12 adenomas or adenocarcinomas and 84 ductal or lobular neoplasms. The protein names were modified to gene names by using the information from TCPA ‘My Protein’ resource.

#### Annotation of mature miRNAs

MiRNA sequencing expression data found in TCGA does not contain information about -3p or -5p arm. This problem has previously been addressed by Kuo et al. (2015) that developed a Python script to interrogate this information. Their idea was applied here using R. The miRNA isoform expression quantification data was utilized from TCGA. A reads per million (rpm) threshold of 1 for calling a gene expressed was applied [51, 52]. Kuo et al. [53] only included the top three expressions of each isoforms for each mature miRNA, whereas here, all values with rpm ≥ 1 were summarized for each miRNA. A ‘gdc sample sheet’, containing information such as file ID and sample ID for each PAAD sample, was also downloaded. The gdc sample sheet was used as input for the R function. For each quantification file, the reads per million (rpm) counts ≥1 were summarized for each MIMA-ID using the plyr package [54]. All samples were merged into one table using merge() function, with option ‘all’ = T, resulting in samples as colnames and MIMAT-IDs in the first column. After merging, data was changed to log2(rpm + 1) and hence all ‘NA’ values were changed to 0. The MIMAT-ID is an identity for each unique miRNA. A file containing information on MIMAT-ID and mature miRNA names (hsa.gff3) was downloaded from miRBase version 22 (www.mirbase.org). The MIMAT-IDs were translated using Perl version 5.18.2 and the miRNA nomenclature file (hsa.gff3). The final output was a table containing the expression levels for each mature miRNA and PAAD sample.

#### Bioinformatics pipeline

The pipeline was built in R version 3.5.1 [55] and consists of miRNA target prediction, correlation analyses and functional enrichment analysis (Fig. 1). The R script, along with instructions for installing and running the pipeline can be accessed through https://emmbor.shinyapps.io/mirfa/.

#### miRNA target prediction

In the implemented pipeline, we included following databases: one experimentally validated database; DIANA-TarBase v7 [25] and two in silico target prediction databases; DIANA-microT-CDS [26] and TargetScan v7.1 [27] for miRNA target prediction. A prediction score threshold was set to 0.7 for DIANA-microT-CDS. DIANA-TarBase v7 (http://diana.imis.athena-innovation.gr/DianaTools/index.php?r=tarbase/index) and DIANA-microT-CDS (http://diana.imis.athena-innovation.gr/DianaTools/index.php) were downloaded. In addition, three different TargetScan v7.1 databases were downloaded; predicted targets for conserved miRNA families, predicted conserved sites for miRNAs and predicted non-conserved sites miRNAs (http://www.targetscan.org/vert_71/). The overlap of identified miRNA target genes from Tarbase, microT-CDS and TargetScan was visualized with Venn diagrams, generated by the VennDiagram package [56].

The downloaded miRNA target prediction databases, along with miRNA, mRNA, and protein expression levels were combined into an sqlite database using sqlite3, called ‘mirna_database.sqlite’ available at https://1drv.ms/u/s!Ap_ICu6nvktNgW6Y68Zkp1HTx0vE, and was queried from the pipeline using RSQLite package [57].

#### Correlation analysis

Correlation analyses were performed to increase the reliability of the predicted miRNA targets by correlating miRNA expression levels to mRNA and protein expression levels of its target genes. Correlations between expression levels of miRNA and mRNA, and miRNA and protein expression levels were performed. The cor() and cor.test() functions were applied using the Pearson’s correlation method. A significance threshold was set at α = 0.05 and multiple testing adjustment using the Benjamini-Hochberg method was performed. Since miRNAs can function as up- or down-regulators of mRNAs, positive and negative correlations were included in subsequent analyses [2, 3].

#### Functional enrichment

Functional enrichment analysis was performed on the correlated miRNA targets [18]. Functional enrichment included enrichment analyses of GO terms and KEGG pathways. The goana() and kegga() functions from the edgeR package were implemented [58, 59]. Criteria of at least 5 genes significantly enriched for a given term and a false discovery rate of 0.05 were used as cutoffs.

#### Shiny web app

To increase the data availability and make miRFA useful for wider community, the pipeline was run for all microRNAs in the TCGA-PAAD dataset (775 miRNAs). The results can be obtained through a shiny application at https://emmbor.shinyapps.io/mirfa/ [17]. Download of data is available at the bottom of each table and Venn diagram plot.

#### Survival analysis

Kaplan-Meier curves were generated using R packages survminer, RTCGA.clinical and survival packages with median as cut-off [60–63]. *P*-values were adjusted for multiple testing using the Benjamini-Hochberg method.

#### Network analysis

Hub genes were identified separately for positively and negatively correlated targets for hsa-miR-885-5p. Positively or negatively correlated gene lists were submitted to the STRING database version 10.5 (https://string-db.org/cgi/input.pl) with *Homo sapiens* as organism and a ‘high confidence’ interaction score (0.7) [64]. The cytohubba plugin [65] was used in Cytoscape version 3.7.0 to show the ranked, top 10 hub genes. ClueGO version 2.5.2 together with CluePedia version 1.5.2 were used to see the overlapping genes between KEGG pathways for positive and negatively correlated targets for hsa-miR-885-5p. Only correlated targets with a Pearson’s correlation coefficient ≥ 0.4 or ≤ − 0.4 were included in the analysis.

## Additional files

Additional file 1: Hsa-miR-22-5p KEGG pathways. Table of enriched KEGG pathways for hsa-miR-22-5p. ‘N’ describes number of genes described for a specific KEGG pathway, ‘DE’ describes number of genes enriched for the pathway and ‘P.DE’ is the *p*-value. (CSV 5 kb)

Additional file 2: Hsa-miR-24-3p KEGG pathways. Table of enriched KEGG pathways for hsa-miR-24-3p. ‘N’ describes number of genes described for a specific KEGG pathway, ‘DE’ describes number of genes enriched for the pathway and ‘P.DE’ is the p-value. (CSV 3 kb)

Additional file 3: Hsa-miR-106b-5p KEGG pathways. Table of enriched KEGG pathways for hsa-miR-106b-5p. ‘N’ describes number of genes described for a specific KEGG pathway, ‘DE’ describes number of genes enriched for the pathway and ‘P.DE’ is the p-value. (CSV 3 kb)

Additional file 4: Hsa-miR-885-5p KEGG pathways. Table of enriched KEGG pathways for hsa-miR-885-5p. ‘N’ describes number of genes described for a specific KEGG pathway, ‘DE’ describes number of genes enriched for the pathway and ‘P.DE’ is the p-value. (CSV 3 kb)

Additional file 5: Significant correlations on mRNA expression level. This table contains all significant correlations on mRNA expression level for all 15 miRNAs. ‘Gene’ is the predicted miRNA target, ‘PCC’ = Pearson’s correlation coefficient, ‘adj_P_value’ = adjusted p-value. (CSV 880 kb)

Additional file 6: **Figure S1.** Venn diagram of DIANA-TarBase v7, DIANA-microT-CDS and TargetScan v7.1 for each miRNA. The R package VennDiagram was used to generate the overlap of identified miRNA target genes. DIANA-TarBase v7 = Tarbase (pink), DIANA-microT-CDS = microT-CDS (blue), TargetScan v.7.1 = TargetScan (grey). **Figure S2.** Overall survival analysis for each miRNA. Kaplan-Meier curves were generated with median as cut-off. Expression=0 is the group that has a value below median and expression=1 is the group that has a value above median. P-values are displayed before multiple hypothesis correction, after multiple hypothesis correction with Benjamini-Hochberg, no miRNA was significant. (DOCX 1105 kb)

## Data Availability

The code for miRFA is available at https://github.com/emmbor/miRFA and our database ‘mirna_database.sqlite’ can be downloaded from https://1drv.ms/u/s!Ap_ICu6nvktNgW6Y68Zkp1HTx0vE. The datasets supporting the conclusions of this article are included within the article and its additional files. Results of all miRNAs detected in TCGA-PAAD can be obtained through our shiny application at https://emmbor.shinyapps.io/mirfa/.

## Abbreviations

AUC: Area under curve
CA 19–9: Carbohydrate antigen 19–9
DIANA: DNA intelligent analysis
GO: Gene ontology
HGNC: HUGO gene nomenclature committee
hsa: *Homo sapiens*
KEGG: Kyoto encyclopedia of genes and genomes
miRFA: miRNA functional analysis
miRNA: microRNA
miRNP: miRNA ribonucleoprotein complex
PAAD: Pancreatic adenocarcinoma
PCC: Pearsons’s correlation coefficient
PDAC: Pancreatic ductal adenocarcinoma
rpm: Reads per million
RPPA: Reverse-phase protein arrays
seq: Sequencing
TCGA: The cancer genome atlas
TCPA: The cancer proteome atlas
TNM: tumor-node-metastasis
