# Supplementary material for: miRFA: an automated pipeline for microRNA functional analysis with correlation support from TCGA and TCPA expression data in pancreatic cancer
Source: BMC Bioinformatics. 2019 Jul 16;20:393. doi: 10.1186/s12859-019-2974-3 (PMC6636046; doi:10.1186/s12859-019-2974-3)
Supplement: Supplementary file 6 — Figure S1. Venn diagram of DIANA-TarBase v7, DIANA-microT-CDS and TargetScan v7.1 for each miRNA. The R package VennDiagram was used to generate the overlap of identified miRNA target genes. DIANA-TarBase v7 = Tarbase (pink), DIANA-microT-CDS = microT-CDS (blue), TargetScan v.7.1 = TargetScan (grey). Figure S2. Overall survival analysis for each miRNA. Kaplan-Meier curves were generated with median as cut-off. Expression=0 is the group that has a value below median and expression=1 is the group that has a value above median. P-values are displayed before multiple hypothesis correction, after multiple hypothesis correction with Benjamini-Hochberg, no miRNA was significant. (DOCX 1105 kb) [file 12859_2019_2974_MOESM6_ESM.docx]

# Supplementary information

## Predicted miRNA targets partially overlap


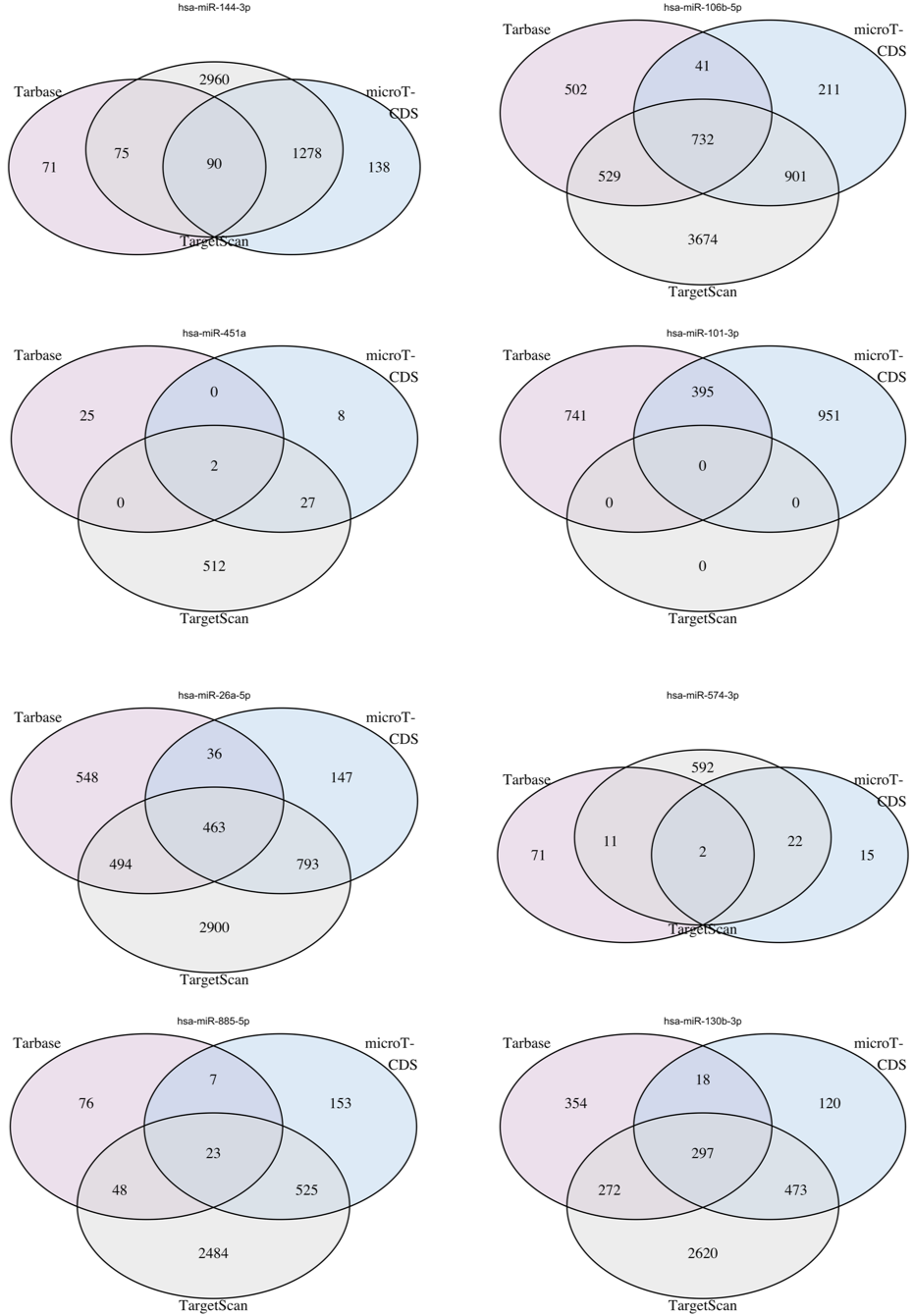


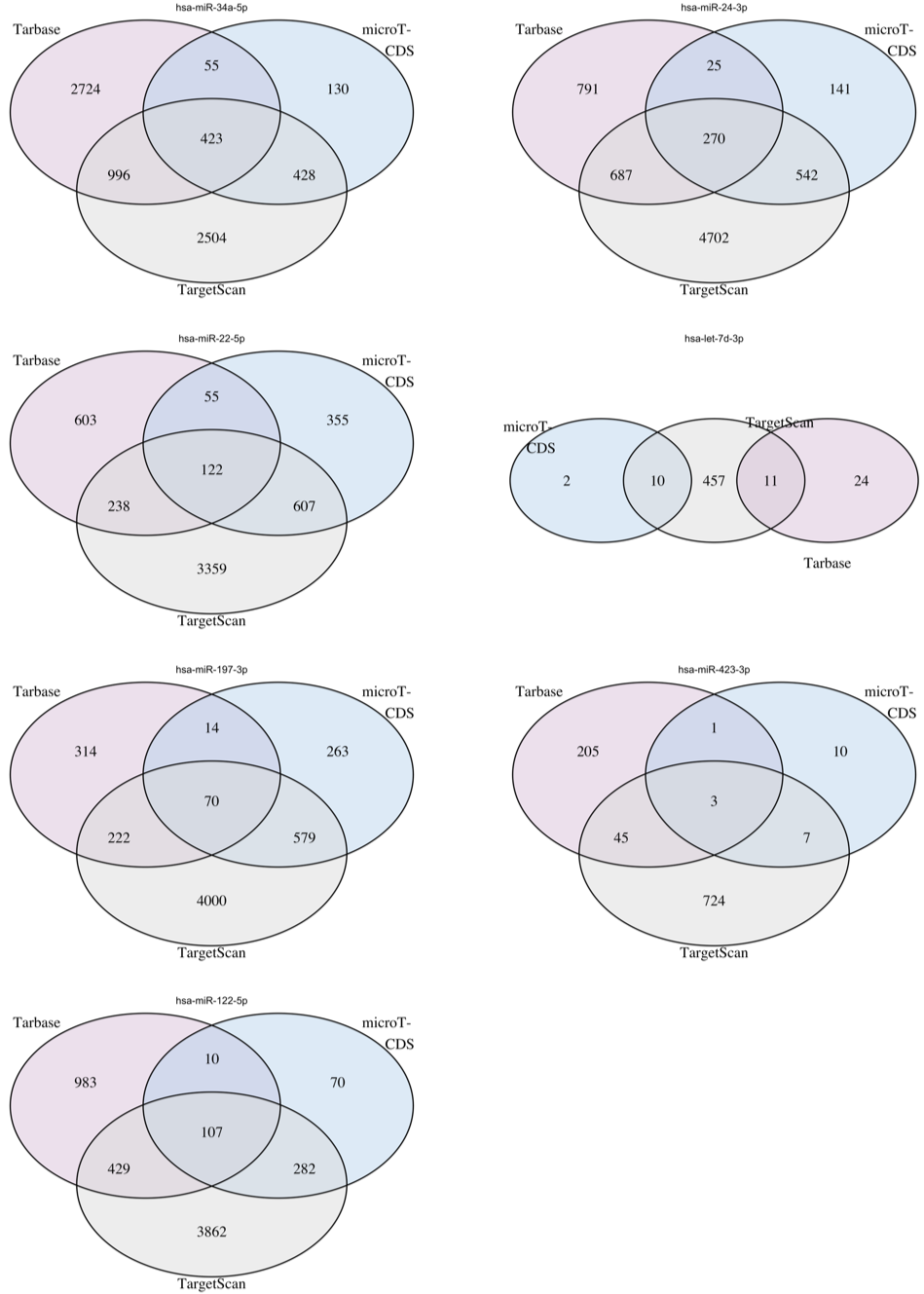


**Figure S1. Venn diagram of DIANA-TarBase v7, DIANA-microT-CDS and TargetScan v7.1 for each miRNA.** The R package VennDiagram was used to generate the overlap of identified miRNA target genes. DIANA-TarBase v7 = Tarbase (pink), DIANA-microT-CDS = microT-CDS (blue), TargetScan v.7.1 = TargetScan (grey).

## Survival analysis


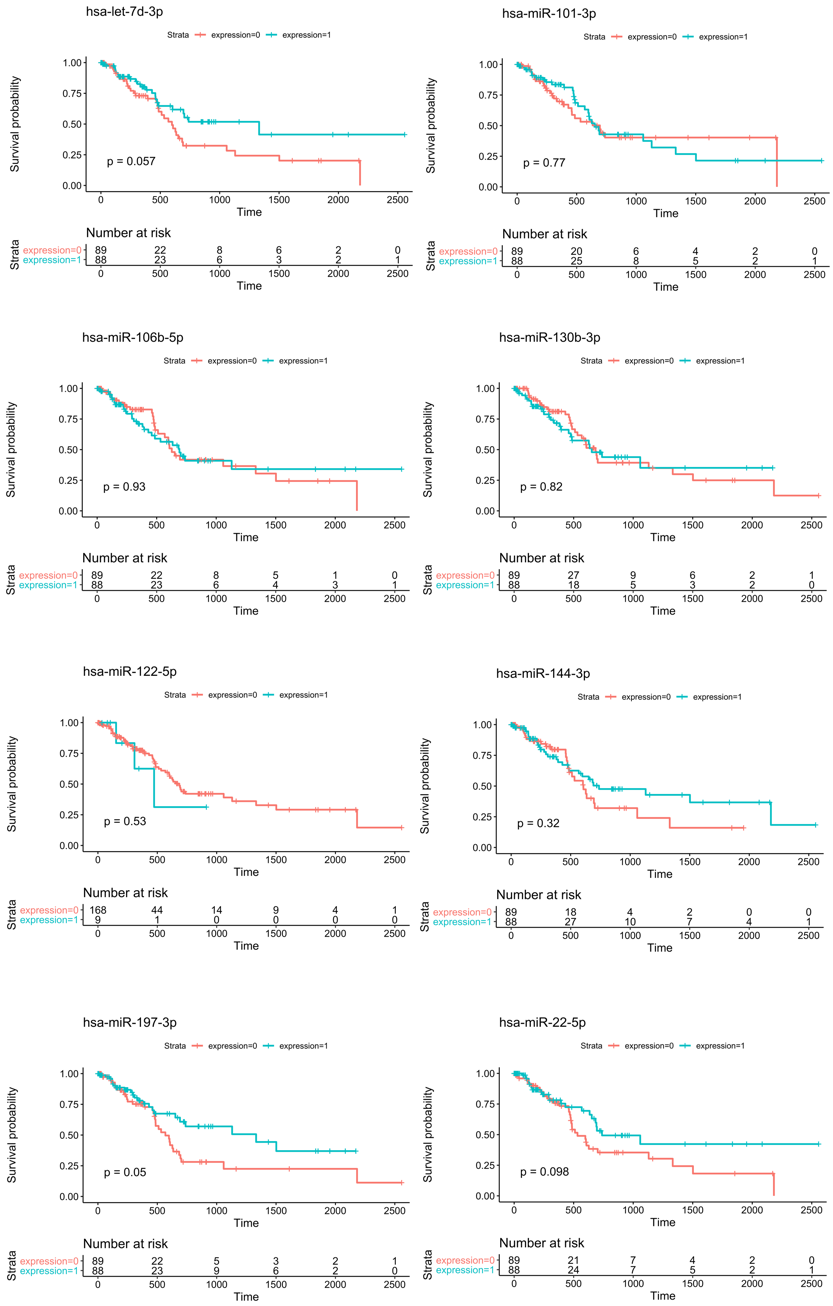

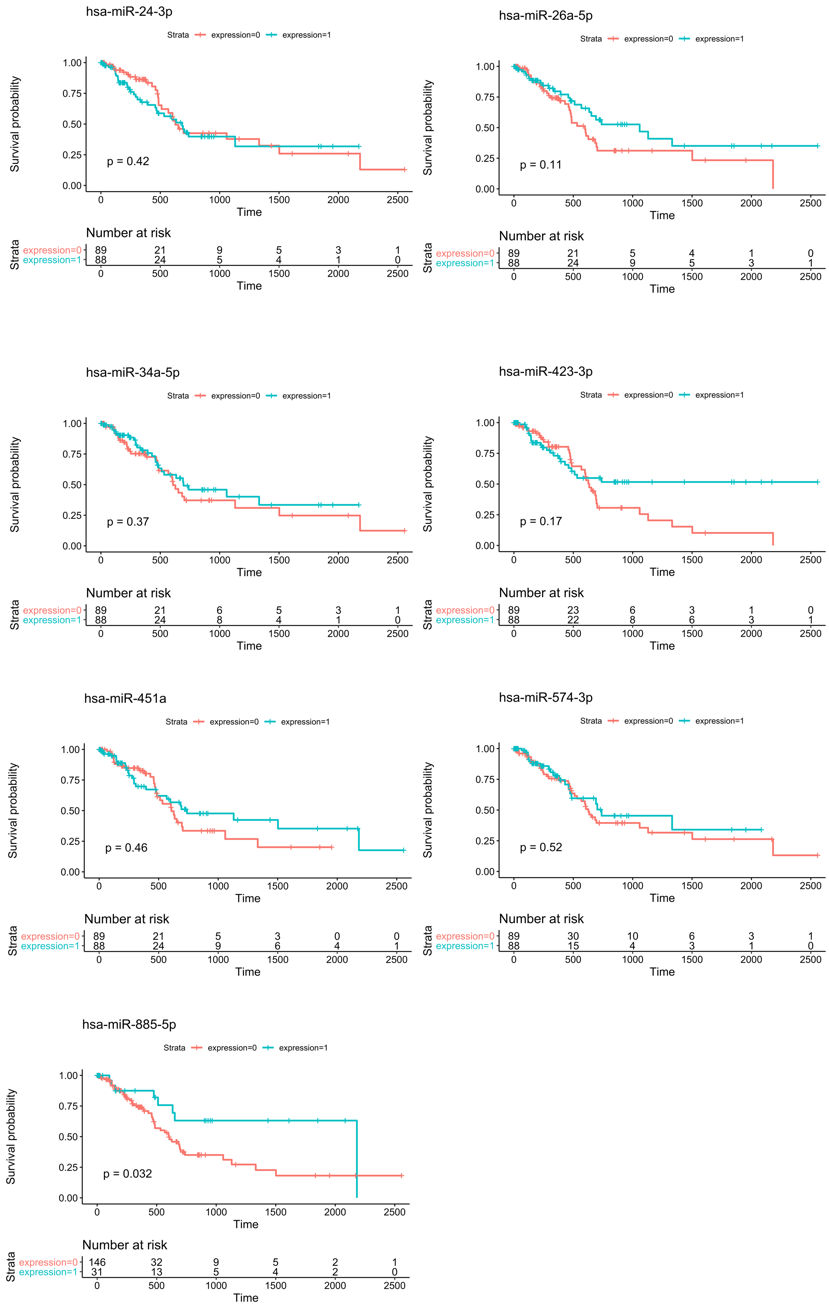


**Figure S2. Overall survival analysis for each miRNA.** Kaplan-Meier curves were generated with median as cut-off. Expression=0 is the group that has a value below median and expression=1 is the group that has a value above median. P-values are displayed before multiple hypothesis correction, after multiple hypothesis correction with Benjamini-Hochberg, no miRNA was significant.
